# A Dynamic Health Assessment Approach for Shearer Based on Artificial Immune Algorithm

**DOI:** 10.1155/2016/9674942

**Published:** 2016-03-31

**Authors:** Zhongbin Wang, Xihua Xu, Lei Si, Rui Ji, Xinhua Liu, Chao Tan

**Affiliations:** ^1^School of Mechatronic Engineering, China University of Mining and Technology, Xuzhou 221116, China; ^2^School of Information and Electrical Engineering, China University of Mining and Technology, Xuzhou 221116, China; ^3^Xuyi Mine Equipment and Materials R&D Center, China University of Mining and Technology, Huai'an 223001, China

## Abstract

In order to accurately identify the dynamic health of shearer, reducing operating trouble and production accident of shearer and improving coal production efficiency further, a dynamic health assessment approach for shearer based on artificial immune algorithm was proposed. The key technologies such as system framework, selecting the indicators for shearer dynamic health assessment, and health assessment model were provided, and the flowchart of the proposed approach was designed. A simulation example, with an accuracy of 96%, based on the collected data from industrial production scene was provided. Furthermore, the comparison demonstrated that the proposed method exhibited higher classification accuracy than the classifiers based on back propagation-neural network (BP-NN) and support vector machine (SVM) methods. Finally, the proposed approach was applied in an engineering problem of shearer dynamic health assessment. The industrial application results showed that the paper research achievements could be used combining with shearer automation control system in fully mechanized coal face. The simulation and the application results indicated that the proposed method was feasible and outperforming others.

## 1. Introduction

Due to the randomicity and complexity of underground geological conditions, assessment of shearer health condition would present the characteristics of complexity, fuzziness, and uncertainty, and this may affect the coal production or even endanger the operator's life. Moreover, because of the poor mining environment and complex component structure of shearer, the shearer operator cannot accurately estimate the working status of shearer, which may lead to some problems of poor coal quality and low mining efficiency. Furthermore, an increasing number of safety accidents in collieries are caused frequently. Therefore, it is necessary to assess the dynamic health condition of shearer which has become a challenging and significant research subject [1].

Depending on the assessment of the health condition of shearer, this can reduce operating trouble and production accident of shearer and improve production efficiency further. In recent years, many researches have brought out some achievement on shearer health condition diagnosis. The multiple fault classifier based on the improved support vector machine theory is used to judge the fault types of coal shearer [2]. In [3], a correct and timely diagnosis mechanism of shearer failures by knowledge acquisition through a fuzzy inference system is provided, which can approximate expert experience. Although many research achievements have been proposed, they have some common shortcomings summarized as follows. Firstly, most research cannot confirm the health degree clearly. Moreover, it costs long diagnosis time and cannot be used in real-time health assessment.

Dynamic health assessment was used in spacecraft primarily in the 1970s. At present, domestic and abroad researchers have worked on the modeling approaches for dynamic health assessment and proposed several solutions. The density-based spatial clustering of applications with noise has been used for bearings' condition monitoring [4], and a novel online method based on dynamic Bayesian networks (DBNs) for the estimation of the SOH of lithium- (Li-) ion batteries has been presented [5], and so on. However, due to the complex component structure and bad working condition, there has not been a health assessment concept on shearer, and this paper tries to present it. In the real mining condition, some key index parameters have a strong relationship with shearer health condition. The relationship is highly nonlinear in nature so that it is hard to develop a comprehensive mathematic model. The current methods and mature assessment systems are hardly satisfied with the shearer health state assessment. In this paper, we try to propose a novel prediction approach for shearer dynamic health assessment to identify the health state during coal mining.

The first mathematical model in artificial immune system was proposed in 1974, which initiated subsequent researches and discussions. Artificial immune system (AIS), as a novel intelligent algorithm method, inspired from the biological immune system, is an effective means for prediction [6–8]. The AIS can acquire learning capability by learning the biological protection principle. According to the above analysis, a novel prediction approach for shearer dynamic health assessment based on artificial immune algorithm is proposed and the assessment system is validated by the sample data of operating parameters from industrial production scene. Moreover, it will prove that artificial immune algorithm is a better tool for classifying due to its classification accuracy than the classifiers based on back propagation-neural network (BP-NN) and support vector machine (SVM) methods later.

The remainder of this paper is organized as follows. Some related works are outlined in Section 2. The key technologies such as system framework, selecting the indicators for shearer dynamic health assessment, and the proposed approach are presented in Section 3.  Section 4 provides a simulation example and an industrial application example for shearer dynamic health assessment based on the proposed approach to specify the application effect. Our conclusions are summarized in Section 5.

## 2. Literature Review

Recent publications relevant to this paper are mainly concerned with two research streams: the dynamic health assessment methods and artificial immune algorithm. In this section, we try to summarize the relevant literatures.

### 2.1. The Dynamic Health Assessment Methods

For the dynamic health assessment problem, lots of research has been done since the last decades. In [9], Zhong-Bin et al. developed a remote monitoring platform of the shearer by using Virtual-Prototype technology to realize the remote monitoring for the shearer in the fully mechanized long-wall coal mining face. In [10], Zhou et al. proposed a novel approach based on the coal floor height variation which is taken as a significant factor and fuzzy optimization theory to improve the implement precision of shearer memory cutting. In [11], P. W. Tse and Y. L. Tse designed an innovative system that is installed in a passenger car or a truck that is running on road and provides instantaneous engine health evaluation and diagnosis. In [12], Black and Winiewicz provided a method and apparatus for internal network device dynamic health monitoring to increase network device availability. In [13], Vichare and Pecht presented the state of practice and the current state of research in the area of electronics prognostics and health management. In [14], Pecht and Jaai presented an assessment of the state of practice in prognostics and health management of information and electronics-rich systems. In [15], Yang et al. proposed an accurate identification of the shearer late underground cutting coal and rock conditions and fault diagnosis by the method of vibration analysis. In [16], Yin et al. designed an embedded health evaluation system to meet the requirement of continuous monitoring of the mine special gear box. In [17], Mascareñas et al. investigated a vibrohaptic human-machine interface for structural health monitoring. In [18], Cerda et al. explored an indirect approach for structural health bridge monitoring allowing for wide, yet cost-effective, bridge stock coverage. In [19], Zubizarreta-Rodriguez and Vasudevan introduced a new multisensor measurement framework for condition monitoring of brushless DC motors (BLDCM) with bearings. In [4], Kerroumi et al. introduced a dynamic classification method inspired by DBSCAN clustering method for machine condition monitoring in general and for bearings in particular. In [5], He et al. presented a novel online method for the estimation of the SOH of lithium- (Li) ion batteries based on dynamic Bayesian networks (DBNs). In [20], Herrmann et al. gave an introduction into the principle of structural health monitoring (SHM), basics of fatigue of fiber resin composite materials, and the possible application of these principles in the automotive industry.

### 2.2. Artificial Immune Algorithm

The artificial immune algorithm was firstly proposed by Farmer in 1986 [21]. It is able to recognize novel shapes without preprogramming based on the capacity of learning, memory, and pattern recognition. In [22], Ishiguro et al. proposed a new decentralized consensus-making system inspired from the biological immune system and an adaptation mechanism that can be used to construct a suitable immune network for adequate action selection. In [23], Tang et al. described a new model of multiple-valued immune network based on biological immune response network. In [24], Abbattista et al. proposed the use of immune network model for designing associative memories. In [25], Deng et al. proposed a fuzzy logic resource allocation and memory cell pruning based artificial immune recognition system (AIRS) to improve the resource allocation mechanism of AIRS and decrease the memory cells. In [26], De Castro and Von Zuben proposed computational implementation of the clonal selection principle that explicitly takes into account the affinity maturation of the immune response. In [27], Chun et al. presented a new method employing the immune algorithm (IA) as the search method for the shape optimization of an electromagnetic device. In [28], Endoh et al. proposed an optimization algorithm based on immune model and applied it to the *n*th agents' travelling salesman problem called *n*-TSP. In [29], Ishiguro et al. proposed a new inference/consensus-making system inspired by immune systems in living organisms, and they apply the proposed method to the behavior arbitration of an autonomous mobile robot as a practical example. In [30], Harmer et al. developed a self-adaptive distributed agent-based defense immune system based on biological strategies within a hierarchical layered architecture. In [31], Pan et al. presented an immune dominance clonal selection multiobjective algorithm based on the artificial immune system to further improve the performance of the optimization algorithm for locomotive secondary spring load adjustment. In [32], Souza et al. presented two new approaches to solving the reconfiguration problem of electrical distribution systems (EDS) using the Copt-aiNet (Artificial Immune Network for Combinatorial Optimization) and Opt-aiNet (Artificial Immune Network for Optimization) algorithms. In [33], Zhang et al. proposed a novel fuzzy hybrid quantum artificial immune clustering algorithm based on cloud model (C-FHQAI) to solve the stochastic problem. In [34], Savsani et al. presented the effect of hybridizing Biogeography-Based Optimization (BBO) technique with artificial immune algorithm (AIA) and Ant Colony Optimization (ACO) in two different ways. In [35], Kuo et al. were dedicated to proposing a cluster analysis algorithm which is integration of artificial immune network (aiNet) and *K*-means algorithm (aiNet*K*).

### 2.3. Discussion

According to the above researches, many health assessment methods, such as density-based spatial clustering and dynamic Bayesian networks, have been applied in the bearings' condition monitoring, network device dynamic health monitoring, and so on. But there are still no relevant studies on the dynamic health assessment methods for shearer. Considering the superiority and universality of artificial immune algorithm, this paper prepares to use this AI algorithm to predict the dynamic health status of shearer. A simulation experiment and an application example are carried out and the proposed approach is proved to be feasible and efficient.

## 3. The Dynamic Health Assessment Approach Based on Artificial Immune Algorithm

### 3.1. The Framework of the Proposed Approach

**Figure 1:**
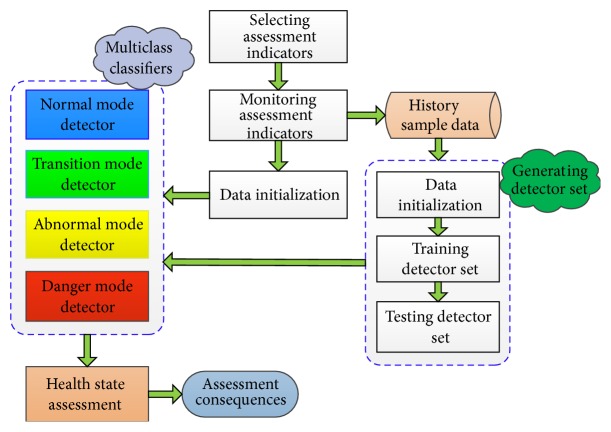
The framework of the proposed approach.

Some real-time running indicators of shearer are usually used to classify the health condition of shearer since the signals can describe its dynamic characteristics. In order to identify the dynamic health status of shearer, the following three processes are required. These processes are assessment indicators selecting, data acquisition and initialization, and multiclass classifiers training and testing. The proposed condition classification approach for shearer dynamic health state is shown in Figure 1. The approach mainly consists of three critical steps: indicators selecting, data initialization, and data training and classification. Firstly, choosing the most effective indicators to assess the health condition of shearer is important since excessive assessment indicators will reduce the impact of main indicators and cause an incorrect result. Then, all the object data in the schema object set are normalized, so the attribute value is within the unit interval [0, 1] and the sample data are divided into four types. Finally, the artificial immune algorithm is used to classify the dynamic health status of shearer.

### 3.2. Selecting the Assessment Indicators

The system of shearer is made up by many subsystems. Establishing a scientific and reasonable evaluation system is the foundation of the health state evaluation for shearer. Depending on the actual operation situation of shearer and referencing other health assessment systems, the assessment consequences for shearer health can be divided into four typical modes: normal mode, transition mode, abnormal mode, and danger mode. The definition of each type of operation is given as follows.

*Normal Mode*. During the working process, the health indicators of shearer change a little and are all in normal range. The shearer works normally.

*Transition Mode*. During the working process, one or two health indicators of shearer have a wide range change occasionally and are not up to the danger line. The shearer works normally and meanwhile the worker of shearer must discover the problem and solve it. 

*Abnormal Mode*. During the working process, some of the health indicators of shearer have a wide range change persistently and are not up to the danger line. The worker of shearer should stop coal production before returning it to normal.

*Dangerous Mode*. During the working process, some of the health indicators of shearer have a sudden change and are up to the danger line. The worker of shearer should stop coal production immediately.

By setting malfunction threshold value depending on operation situation, four modes of shearer health situation decrease progressively. Four different health modes can guide coal worker adopting corresponding operation, respectively.

**Figure 2:**
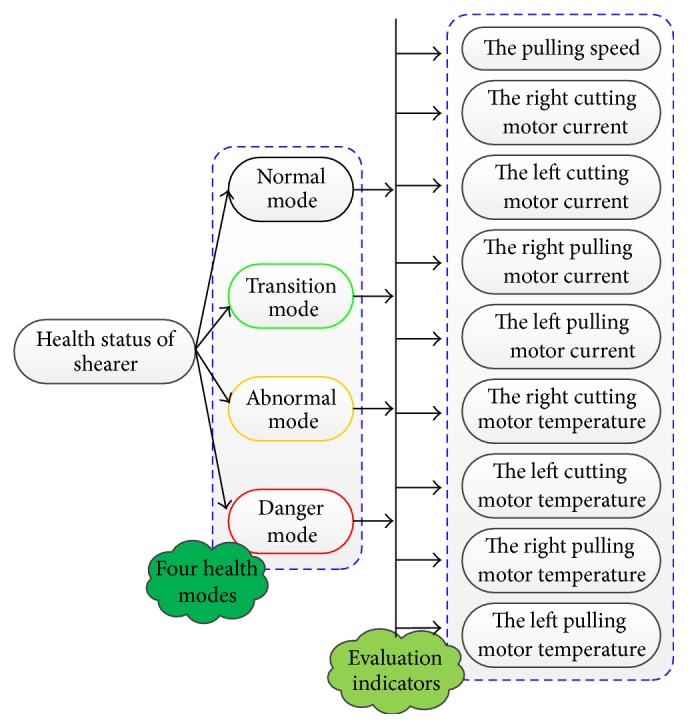
The indicators of dynamic health assessment model for shearer.

The system of shearer is made up by many subsystems. The data from historical recording and real-time monitoring of the subsystems reflect the health status of shearer more or less. However, in practical application, we must choose the most effective indicators to assess the health situation of shearer and eliminate subordinate indicators, as excessive assessment indicators will reduce the impact of the main indicators, causing an incorrect result. According to the expert experience and actual working condition of shearer, the dynamic health condition depends on the real-time monitoring data. In this paper, the key content is the real-time health assessment of shearer. Thus, to assess the dynamic health situation of shearer, we choose nine real-time running indicators: the pulling speed *p*_1_, the right cutting motor current *p*_2_ and the left cutting motor current *p*_3_, the right pulling motor current *p*_4_ and the left pulling motor current *p*_5_, the right cutting motor temperature *p*_6_ and the left cutting motor temperature *p*_7_, and the right pulling motor temperature *p*_8_ and the left pulling motor temperature *p*_9_. There are test data showing that the pulling speed has a mapping relation with working load of shearer. Monitoring the change of the pulling speed can reflect the working load in a degree. Moreover, as the most important information on judging shearer operating state, the cutting motor current and the pulling motor current can be influenced by the pulling speed, the cutting drum height, the working load, the coal-rock characteristic, and so on. The difference between the two currents is that the cutting motor current has a direct proportion with working load, while the pulling motor current can comprehensively characterize the pulling resistance. Finally, the slow change of the cutting motor temperature and the pulling motor temperature can represent the general state of working load and pulling load over a period of time. The nine indicators can reflect the shearer operation state clearly. The assessment indicators of dynamic health assessment model for shearer can be shown in Figure 2.

### 3.3. Defining Detectors Set

**Figure 3:**
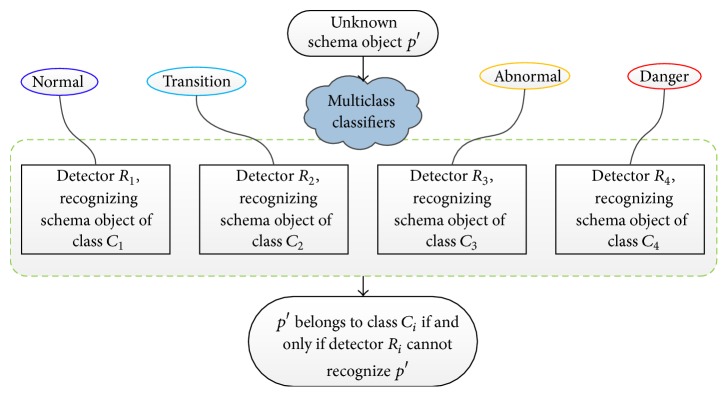
Immune classifier model of dynamic health assessment for shearer.

According to the nine indicators of shearer dynamic health assessment approach defined above, we can determine the unknown schema object *p*′ for nine-dimensional attribute space, shown in detail in Definition 1. Corresponding to the four modes of shearer dynamic health assessment consequences, multiclass classifiers are constituted by four detectors: normal mode (*C*_1_), transition mode (*C*_2_), abnormal mode (*C*_3_), and danger mode (*C*_4_). Any one of the non-self-class objects (the schema object of classes *C*_1_,…, *C*_*i*−1_, *C*_*i*+1_,…, *C*_4_) can be recognized by the *i*th detector (*R*_*i*_), excepting the self-class object (the schema object of class *C*_*i*_). In other words, each detector only cannot recognize the corresponding class object of particular assessment consequence mode. The immune classifier model of dynamic health assessment for shearer is shown in Figure 3.

Before establishing the dynamic health assessment model based on artificial immune algorithm, the related definitions of multiclass classifiers are given as follows.

**Definition 1 .**

Each schema object can be represented as a *k*-dimensional vector (*p*, *c*) = (*p*_1_, *p*_2_,…, *p*_*k*_, *c*), and *P* is data set of the schema object, *p* ∈ *P*, where *k* = 9 is the number of attributes of the schema object and *c* is attribute class of schema object.

**Definition 2 .**

Detectors set *R* can recognize all the certain type data in the schema object set. Each member of the detectors set is called receptor, marked as *r*, (*r*, *c*) = (*r*_1_, *r*_2_,…, *r*_*k*_, *c*). The receptor has similar structure to the schema object.

**Definition 3 .**

Receptor can recognize any one of the schema objects of a certain type. The degree of similarity between receptor and schema object can measure affinity:

(1) affinityr,p=1−Dr,pkDr,p=∑i=1kri−pi2,

where *R* is detectors set, *r* ∈ *R*.

*D*(*r*, *p*) is the Euclidean distance of schema object *p* and detector *r*.

The function value of affinity lies between 0 and 1. The more similar the value between schema object *p* and detector *r*, the greater the function value of affinity.

**Definition 4 .**

∂_selection_ is a choosing threshold for selected receptor and the value lies between 0 and 1. The choosing threshold is very important, as it selects which receptors should be removed from detectors set in the step of training.

**Definition 5 .**

∂_detection_ is a testing threshold for detectors set. The value of testing threshold is the key to correctly classify detectors, as it decides which detectors should be activated in the step of testing.

### 3.4. Establishing the Assessment Model

In this section, the flows for establishing the dynamic health assessment model based on artificial immune algorithm are provided in detail. It mainly includes three steps.

#### 3.4.1. Data Initialization

This step can be regarded as a data preprocessing stage. Each schema object is represented as a 9-dimensional vector (*p*, *c*) = (*p*_1_, *p*_2_,…, *p*_9_, *c*), *p* ∈ *P*. All object data of assessment indicators in the schema object set are normalized, so the attribute value is within the unit interval [0,1] and *p*_*i*_ ∈ (0,1). To correspond to the four patterns of shearer dynamic health assessment consequences, the training data of schema object set are divided into four types: normal mode (*D*_1_), transition mode (*D*_2_), abnormal mode (*D*_3_), and danger mode (*D*_4_).

#### 3.4.2. Training Detectors Set

The purpose of training stage is generating an effective detector for each schema object. The steps for generating detector are given as follows.

**Step 1 .**

Take preprocessed *D*_*i*_ as self-data set *D*_self_, *D*_self_ = *D*_*i*_, so non-self-data set *D*_nonself_ was made up by the other preprocessed data sets, *D*_nonself_ = {*D*_1_ ∪ ⋯∪*D*_*i*−1_ ∪ *D*_*i*+1_ ∪ ⋯∪*D*_*N*_}. Initial detector *R*_*i*_ is empty, *R*_*i*_ = *∅*.

**Step 2 .**

Generate random alternative detectors set *R*′.

**Step 3 .**

Calculate affinity between *R*′ and *D*_self_; if affinity(*p*_*i*_, *r*_*j*_) > ∂_selection_, delete *r*_*j*_ from *R*′ (negative selection).

**Step 4 .**

Calculate affinity between *R*′ and *D*_nonself_; if affinity(*p*_*i*_, *r*_*j*_) < ∂_selection_, delete *r*_*j*_ from *R*′ (positive selection).

**Step 5 .**

Delete the individual from *D*_nonself_, if it can be recognized by *R*′.

**Step 6 .**

If *D*_nonself_ = *∅*, detector *R*_*i*_ is accomplished, and iteration is finished, *R*_*i*_ = *R*_*i*_ ∪ *R*′; otherwise, turn back to Step 2.

**Figure 4:**
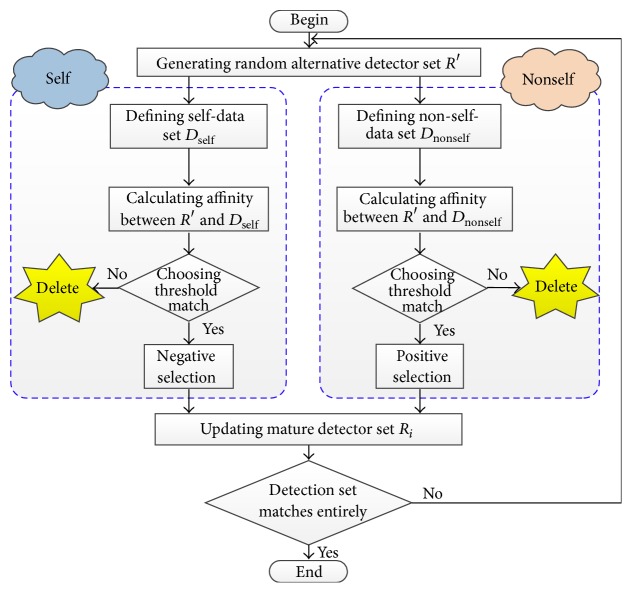
The generation process of a detector.

Using the same negative selection algorithm of generating detector, repeat calculation four times from class object *C*_1_ to class object *C*_4_ to all detector sets until every detector can distinguish self-class object and non-self-class object. The generation process of a detector is shown in Figure 4.

#### 3.4.3. Testing Detectors Set

**Figure 5:**
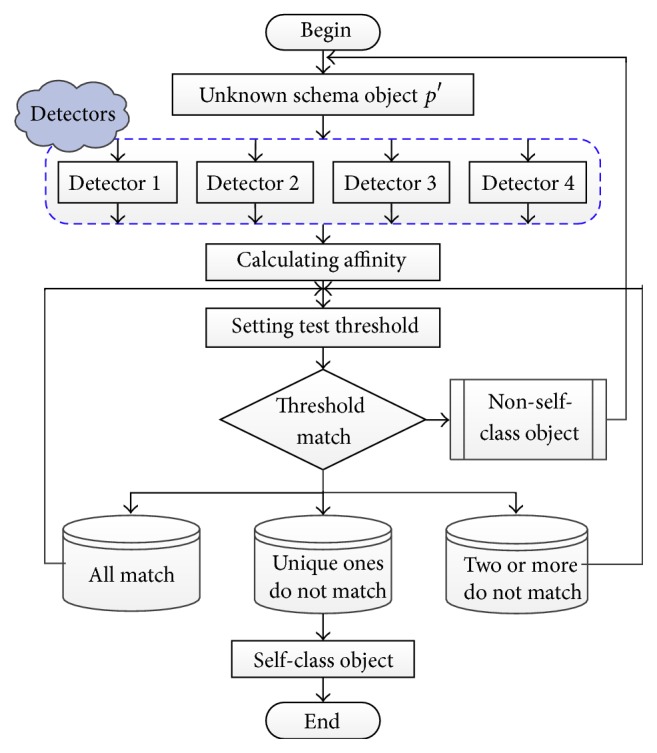
Flowchart of negative selection test for a new sample.

To distinguish self-class object and non-self-class object, all detectors are used for circulatory elimination for new sample in testing step. The flowchart of negative selection test for a new sample is shown in Figure 5.

*p*′ is sample data of unknown schema object for inputting. Then, calculate the value ∂_detection_ between *p*′ and all detectors (*R*_1_ ~ *R*_*i*_). If data *p*′ can be recognized by detector *R*_*m*_(1 ≤ *m* ≤ *i*), data *p*′ does not belong to the schema object *C*_*m*_. Repeat this process until all detectors are tested. The final consequence will be one of the following cases.

**Case 1 .**

If schema object *p*′ only cannot be recognized by detector *R*_*i*_, then schema object *p*′ belongs to class object *C*_*i*_.

**Case 2 .**

If schema object *p*′ cannot be recognized by two or more detectors, set a new test threshold value. Calculate the value ∂_detection_ between *p*′ and nonactivated detectors. Repeat this process until only one detector remained nonactivated. Then, schema object *p*′ belongs to the corresponding schema object of the last nonactivated detector.

**Case 3 .**

If schema object *p*′ can be recognized by all detectors, set a new test threshold value. Calculate the value ∂_detection_ between *p*′ and all detectors. Repeat this process until only one detector remained nonactivated. Then, schema object *p*′ belongs to the corresponding schema object of the last nonactivated detector.

## 4. Simulation Examples and Application

### 4.1. Simulation Examples

In this section, some simulation examples were put forward to verify the feasibility and efficiency of the proposed approach.

The sample data were acquired from the shearer in 22210 fully mechanized coal face of Zhong Ping Energy Chemical Group No. 6 Mine. Depending on the assessment model of prediction approach of shearer dynamic health assessment, the acquired data were normalized so that the object data were represented as a 9-dimensional vector, (*p*, *c*) = (*p*_1_, *p*_2_,…, *p*_9_, *c*), *p* ∈ *P*. The data in the schema object set was initialized, so the attribute value was within the unit interval [0,1], *p*_*i*_ ∈ (0,1). The training data of schema object set were divided into four types: normal mode (*D*_1_), transition mode (*D*_2_), abnormal mode (*D*_3_), and danger mode (*D*_4_). As shown in Table 1, 1000 groups of data were randomly chosen to train the detectors set and the last 200 groups were used to test the classification performance of the trained detectors.

After the assessment model based on artificial immune algorithm was trained, the multiclass classifiers of the assessment system were constituted by four detectors, and each detector only could not recognize corresponding class object of particular assessment consequence mode. Actually, if the input schema object only could not be recognized by one detector, then the schema object belongs to this class object.

**Figure 6:**
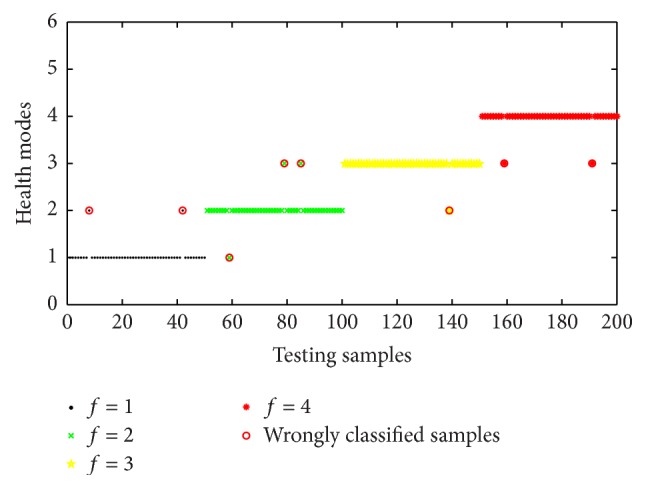
Classification results of the classifier based on artificial immune algorithm.

After the training phase, an assessment system could be obtained. In order to verify the accuracy of the model, the remaining 200 samples were utilized to test its performance. The prediction consequence was given as in Figure 6. As shown in Figure 6, only eight testing samples were misclassified and circled in red. The ordinate values 1, 2, 3, and 4 corresponded to four assessment consequences of shearer dynamic health modes, and identification accuracies of four detectors were 96%, 94%, 98%, and 96%, respectively. The overall average classification accuracy was 96%, which satisfied the engineering requirement. The testing results indicated that the proposed approach performed with lower deviation and could be applied in the assessment of shearer dynamic health.

**Figure 7:**
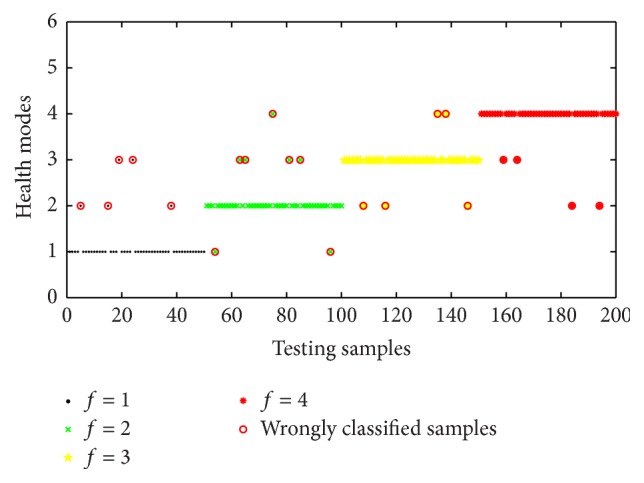
Classification results of the classifier based on BP-NN.

In order to indicate the meliority of assessment model based on artificial immune algorithm, the assessment model based on back propagation-neural network (BP-NN) and support vector machine (SVM) was provided to solve the problem of the above example. The training samples and testing samples were the same. The configurations of simulation environment for three algorithms were uniform and the relevant parameters were in common with the above example. The prediction consequence of the assessment model based on BP-NN was given as in Figure 7. As shown in Figure 7, twenty-one testing samples were misclassified and some samples had a large deviation to real situations. The classification accuracies for four health modes were 90%, 86%, 90%, and 92%, and overall average classification accuracy was 89.5%.

**Figure 8:**
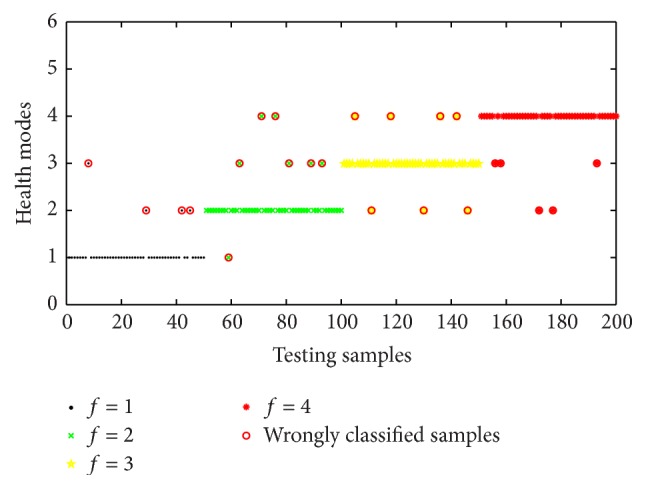
Classification results of the classifier based on SVM.

The prediction consequence of the assessment model based on SVM was given as in Figure 8. As shown in Figure 8, twenty-three testing samples were misclassified and some samples had a large deviation to real situations. The classification accuracies for four health modes were 92%, 86%, 88%, and 90%, and overall average classification accuracy was 88.5%. It was observed that the proposed method had a better classification capability and performance than the competing methods. With the benefits of artificial immune algorithm in uncertain fields, the proposed classifier could obtain higher classification accuracy than single BP-NN and SVM classifiers.

### 4.2. Further Discussion

In order to further compare and analyze the overall performance of SVM, BP-NN, and AI, the same 1200 samples are experimented with. In this example, a certain number of samples, denoted by training size (*T*_size_), are randomly selected from the data as the training samples and 200 samples are randomly selected from the remaining 1200 − *T*_size_ samples as the testing samples. Each learning algorithm is then trained and tested 200 times and the classification error rate is recorded as the final result. In this study, the training size of the example varies over *T*_size_ = 100,120,140, 160,…, 1000. That is to say, we run several trials over the algorithms with training size ranging from 100 to 1000. The classification error rate *m*/*n* (where *m* is the classification error times and *n* is the total test times) is chosen as the metric to express the result as a proportion of the optimal solution.

**Figure 9:**
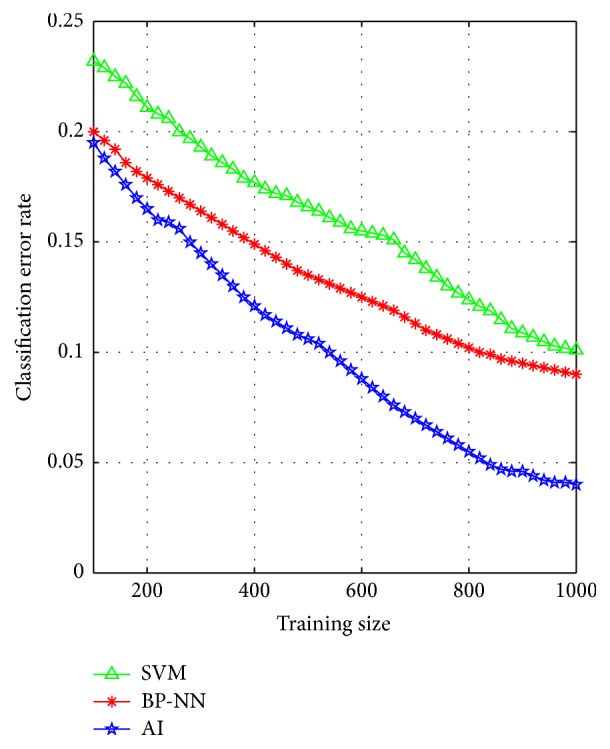
The changes of classification error rate with different training sizes.

Figure 9 plots the means of this metric (classification error rate) for each trial as a function of problem size *T*_size_. It can be seen that for all trials the classification error rate decreases nonlinearly with *T*_size_ and the classification accuracy of AI outperforms BP-NN and SVM for all *T*_size_.

From Figure 9, it is obvious that the classification error rate descent velocity of AI is the fastest across different training sizes and owns stronger generalization ability than BP-NN and SVM regardless of the training size. What is more, the classification accuracy of AI is more supernal and robust. Therefore, the AI algorithm can obtain a relatively high accuracy to provide an effective support tool for dynamic health assessment for shearer.

### 4.3. Industrial Application

**Figure 10:**
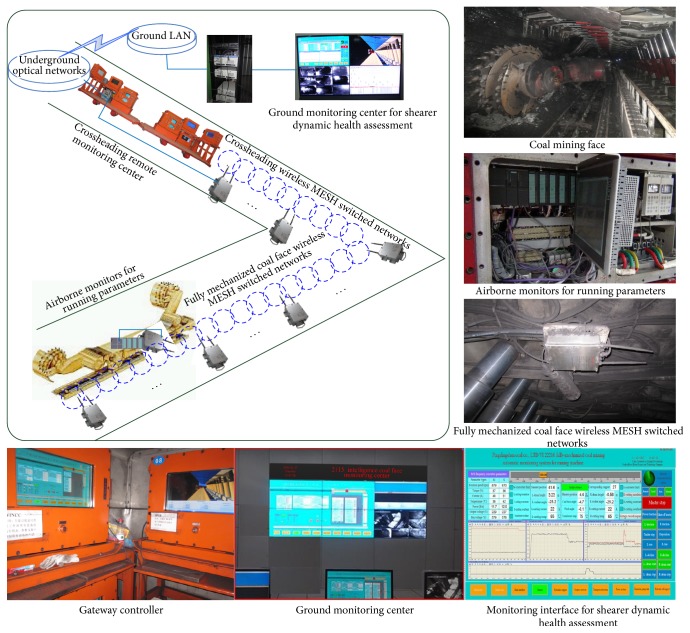
Hardware construction in fully mechanized coal face.

In this section, a system based on the proposed approach had been developed and applied in the field of shearer dynamic health assessment as shown in Figure 10.

As Figure 10 showed, the “gateway controller” and “ground monitoring center” were used to control and monitor the shearer running parameters. The system based on the proposed approach was uploaded into the gateway controller. The pulling speed, the left cutting motor current, the right cutting motor current, the left pulling motor current, the right pulling motor current, the left cutting motor temperature, the right cutting motor temperature, the left pulling motor temperature, and the right pulling motor temperature were collected every 1 Hz from the shearer controller and the collected data were transmitted to the gateway controller. Then, the changes of shearer dynamic health assessment consequence were identified and showed on “monitoring interface for shearer dynamic health assessment.”

**Figure 11:**
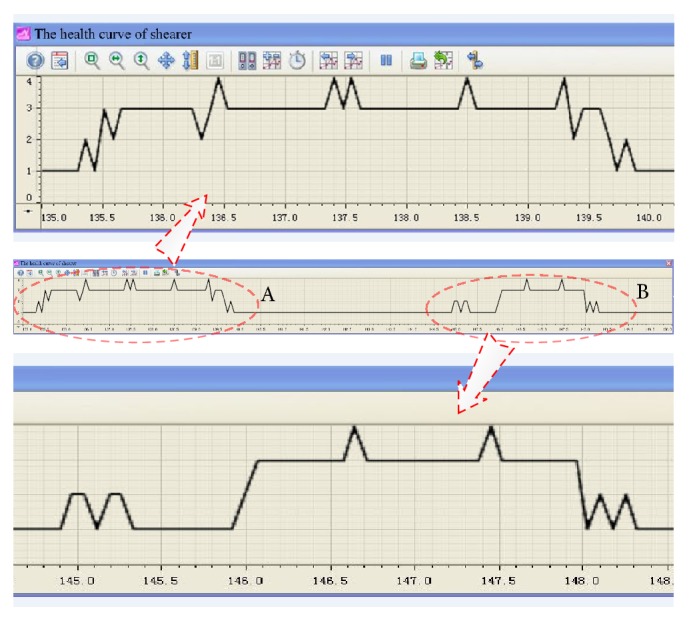
The dynamic health assessment curve of shearer based on the proposed system.

**Figure 12:**
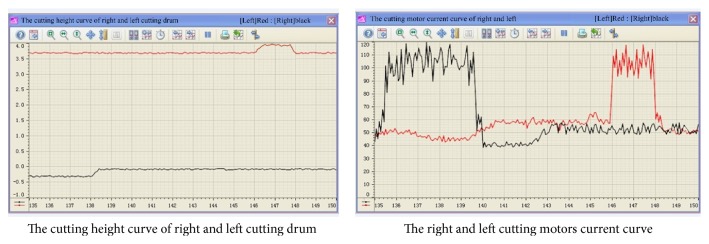
The operational parameters curve of shearer with the manual operation.

In order to illustrate the application effect of the proposed approach, the shearer was running in fully mechanized coal face from 135.0 m to 150.0 m by the manual operation. The dynamic health assessment curve based on the proposed classifier was shown in Figure 11, and the ordinate values of 1, 2, 3, and 4 denoted four health classes: normal mode, transition mode, abnormal mode, and danger mode. The curve showed two obvious changes in segments A and B. The ordinate values were leaped from 1 to 3, which means that the shearer health status had a sudden change from normal mode to abnormal mode. The changes of some operational parameters were plotted in Figure 12. The cutting motor current had a noticeable increase when the right cutting drum cut the floor between 135.0 m and 138.5 m and the left cutting drum cut the roof between 146.0 m and 148.0 m. The results of shearer dynamic health assessment based on the proposed system were almost completely consistent with the actual cutting status of shearer.

## 5. Conclusions and Future Work

The main contribution of this paper was that a methodology based on artificial immune algorithm for the assessment of shearer dynamic health status was presented. The detailed flows for the proposed approach were described, including three critical steps, that is, assessment indicators selecting, data acquisition and initialization, and multiclass classifiers training and testing. In order to verify the feasibility and efficiency of the proposed approach, a simulation example was provided and some comparisons with other algorithms were carried out. The simulation results showed that the proposed approach was outperforming others. Finally, the proposed approach was applied to an engineering problem of shearer dynamic health assessment. The industrial application results showed that the paper research achievements could be used combining with shearer automation control system in fully mechanized coal face and had obvious effectiveness on reducing operating trouble and production accident of shearer and improving coal production efficiency further. The artificial immune algorithm could obtain a relatively high accuracy to provide an effective support tool for dynamic health assessment for shearer.

In future studies, the authors plan to investigate some improvements for the proposed approach. Possible improvements may include the combination of artificial immune algorithm with other intelligent algorithms for better performance. In addition, the applications of the proposed approach in dynamic health assessment domain are worth further study from the authors.

## Figures and Tables

**Table 1:** Normalized data of pattern objects for shearer.

Number	*P*_1_	*P*_2_	*P*_3_	*P*_4_	*P*_5_	*P*_6_	*P*_7_	*P*_8_	*P*_9_	Categories
1	0.226	0.515	0.485	0.559	0.539	0.455	0.568	0.298	0.338	*D*_1_
2	0.194	0.540	0.485	0.557	0.535	0.455	0.565	0.287	0.328	*D*_1_
3	0.168	0.577	0.494	0.486	0.480	0.458	0.565	0.291	0.335	*D*_1_
4	0.167	0.562	0.485	0.513	0.496	0.458	0.565	0.290	0.338	*D*_1_
⋮	⋮	⋮	⋮	⋮	⋮	⋮	⋮	⋮	⋮	⋮
185	0.257	0.509	0.494	0.461	0.489	0.458	0.565	0.296	0.343	*D*_1_
186	0.258	0.519	0.506	0.471	0.497	0.455	0.565	0.295	0.343	*D*_1_
187	0.000	0.485	0.497	0.444	0.432	0.458	0.570	0.309	0.329	*D*_2_
188	0.227	0.503	0.488	0.417	0.422	0.458	0.568	0.281	0.327	*D*_1_
⋮	⋮	⋮	⋮	⋮	⋮	⋮	⋮	⋮	⋮	⋮
354	0.350	0.519	0.491	0.459	0.450	0.458	0.565	0.284	0.329	*D*_1_
355	0.858	0.552	0.821	0.531	0.704	0.458	0.570	0.301	0.350	*D*_3_
356	0.000	0.485	0.497	0.444	0.432	0.458	0.570	0.309	0.329	*D*_2_
357	0.408	0.522	0.497	0.455	0.445	0.458	0.568	0.280	0.327	*D*_1_
⋮	⋮	⋮	⋮	⋮	⋮	⋮	⋮	⋮	⋮	⋮
587	0.000	0.485	0.497	0.444	0.432	0.458	0.570	0.309	0.329	*D*_2_
588	0.773	0.556	0.990	0.659	0.653	0.458	0.568	0.304	0.348	*D*_4_
589	0.850	0.503	0.907	0.630	0.619	0.458	0.568	0.301	0.348	*D*_4_
590	0.854	0.540	0.861	0.587	0.635	0.458	0.568	0.303	0.349	*D*_3_
⋮	⋮	⋮	⋮	⋮	⋮	⋮	⋮	⋮	⋮	⋮
753	0.943	0.506	0.509	0.816	0.783	0.461	0.570	0.311	0.359	*D*_3_
754	0.950	0.509	0.707	0.630	0.619	0.458	0.568	0.301	0.348	*D*_4_
755	0.000	0.481	0.497	0.445	0.432	0.458	0.570	0.313	0.352	*D*_2_
756	0.000	0.494	0.497	0.449	0.431	0.458	0.570	0.313	0.352	*D*_2_
⋮	⋮	⋮	⋮	⋮	⋮	⋮	⋮	⋮	⋮	⋮
893	0.042	0.491	0.497	0.386	0.329	0.458	0.570	0.294	0.339	*D*_2_
894	0.151	0.494	0.491	0.405	0.419	0.458	0.570	0.290	0.342	*D*_2_
895	0.950	0.494	0.503	0.708	0.724	0.461	0.570	0.296	0.354	*D*_3_
896	0.399	0.488	0.491	0.389	0.393	0.458	0.570	0.288	0.338	*D*_2_
⋮	⋮	⋮	⋮	⋮	⋮	⋮	⋮	⋮	⋮	⋮
1072	0.950	0.506	0.506	0.621	0.783	0.461	0.570	0.297	0.358	*D*_3_
1073	0.948	0.506	0.500	0.695	0.658	0.461	0.570	0.298	0.358	*D*_3_
1074	0.950	0.509	0.707	0.630	0.619	0.458	0.568	0.301	0.348	*D*_4_
1075	0.854	0.506	0.920	0.587	0.635	0.458	0.568	0.303	0.349	*D*_4_
⋮	⋮	⋮	⋮	⋮	⋮	⋮	⋮	⋮	⋮	⋮
1200	0.347	0.500	0.475	0.535	0.534	0.464	0.527	0.298	0.326	*D*_1_
